# Impact of migraine on fibromyalgia symptoms

**DOI:** 10.1186/s10194-016-0619-8

**Published:** 2016-03-22

**Authors:** Maria Adele Giamberardino, Giannapia Affaitati, Paolo Martelletti, Claudio Tana, Andrea Negro, Domenico Lapenna, Martina Curto, Cosima Schiavone, Luisa Stellin, Francesco Cipollone, Raffaele Costantini

**Affiliations:** Fibromyalgia and Headache Center, Geriatrics Clinics, Department of Medicine and Science of Aging and Ce.S.I., “G. D’Annunzio” University of Chieti, via dei Vestini s.n., 66100 Chieti, Italy; Department of Clinical and Molecular Medicine, Regional Referral Headache Centre, “Sant’Andrea” Hospital, “Sapienza” University, Rome, Italy; Internal Medicine Unit, Guastalla Hospital, AUSL Reggio Emilia, Reggio Emilia, Italy; Department of Medicine and Science of Aging, “G. D’Annunzio” University of Chieti, Chieti, Italy; Institute of Surgical Pathology, “G. D’Annunzio” University of Chieti, Chieti, Italy

**Keywords:** Migraine, Fibromyalgia, Pain thresholds, Tender points, Hyperalgesia, Central sensitization

## Abstract

**Background:**

Fibromyalgia (FMS) and high frequency episodic/chronic migraine (M) very frequently co-occur, suggesting common pathophysiological mechanisms; both conditions display generalized somatic hyperalgesia. In FMS-M comorbidity we assessed if: a different level of hyperalgesia is present compared to one condition only; hyperalgesia is a function of migraine frequency; migraine attacks trigger FMS symptoms.

**Methods:**

Female patients with fibromyalgia (FMS)(n.40), high frequency episodic migraine (M1)(n.41), chronic migraine (M2)(n.40), FMS + M1 (n.42) and FMS + M2 (n.40) underwent recording of: −electrical pain thresholds in skin, subcutis and muscle and pressure pain thresholds in control sites, −pressure pain thresholds in tender points (TePs), −number of monthly migraine attacks and fibromyalgia flares (3-month diary). Migraine and FMS parameters were evaluated before and after migraine prophylaxis, or no prophylaxis, for 3 months with calcium-channel blockers, in two further FMS + H1 groups (n.49, n.39). 1-way ANOVA was applied to test trends among groups, Student’s *t*-test for paired samples was used to compare pre and post-treatment values.

**Results:**

The lowest electrical and pressure thresholds at all sites and tissues were found in FMS + M2, followed by FMS + H1, FMS, M2 and M1 (trend: *p* < 0.0001). FMS monthly flares were progressively higher in FMS, FMS + M1 and FMS + M2 (*p* < 0.0001); most flares (86–87 %) occurred within 12 h from a migraine attack in co-morbid patients (*p* < 0.0001). Effective migraine prophylaxis vs no prophylaxis also produced a significant improvement of FMS symptoms (decreased monthly flares, increased pain thresholds)(0.0001 < *p* < 0.003).

**Conclusions:**

Co-morbidity between fibromyalgia and migraine involves heightened somatic hyperalgesia compared to one condition only. Increased migraine frequency – with shift towards chronicity – enhances both hyperalgesia and spontaneous FMS pain, which is reversed by effective migraine prophylaxis. These results suggest different levels of central sensitization in patients with migraine, fibromyalgia or both conditions and a role for migraine as a triggering factor for FMS.

## Background

Fibromyalgia Syndrome (FMS) is a chronic pain condition whose prevalence in the general population ranges from 4 to 7 %, with a net female predominance [1, 2]. According to the 1990 ACR criteria, the syndrome is diagnosed if 2 main conditions are fulfilled: 1) presence of diffuse musculoskeletal pain of at least 3 months’ duration and 2) positivity of at least 11 out of 18 predetermined body sites (9 symmetrical) called tender points (TePs), i.e., tenderness for an applied standard pressure of 4 kg-f exerted either manually or via a pressure algometer [3]. These criteria were revised in 2010 [4], with preliminary new criteria no longer taking into account the TeP count, while introducing more clinical requisites in addition to the diffuse muscle pain, e.g., the presence of sleep disorders, affective dysfunction, headache or visceral pains [5–9]. FMS has, indeed, a high degree of comorbidity with a number of other medical conditions, among which headache, especially with an elevated number of attacks or chronic, is particularly frequent [10, 11]. Though tension-type headache is the most prevalent type in FMS, there is also a high co-occurrence between the syndrome and migraine [12]. A recent large epidemiologic study, in fact, evidenced a 55.8 % prevalence of migraine among fibromyalgia patients [13], while other studies showed the prevalence of fibromyalgia in migraine patients to be over 30 % [14, 15]. Although the pathophysiology of FMS is still incompletely known, a crucial role in the syndrome is believed to be played by central mechanisms of sensitization, secondary to an imbalance of neuromediators involved in nociceptive transmission/control in the Central Nervous System (CNS), in genetically predisposed subjects [16-18]. Clinical evidence of sensitization is provided by the generalized decrease in pain thresholds to different modalities of stimuli at somatic level not only in spontaneous painful areas but also in control, nonpainful sites, which has been widely documented in the syndrome [6, 19, 20]. High frequency and chronic headache have also been found to display increased sensitivity to pain in somatic areas outside the cephalic region, although to a lesser extent with respect to fibromyalgia [21–28]. These observations have raised the question as to whether the association of FMS with headache involves higher degrees of central sensitization with respect to one condition only. Clinical observations also report that FMS patients with concurrent headache, particularly migraine, often present an exacerbation of their typical FMS symptomatology in concomitance with or immediately subsequent to a headache attack, suggesting that headache may represent a triggering factor for fibromyalgia pain. However, in spite of the high degree of co-occurrence between headache and fibromyalgia, no systematic studies appear to have been conducted so far to evaluate the implications of this co-morbidity not only for the spontaneous FMS symptoms but also for the general sensory asset of the patients. This kind of study is instead important not only to better investigate the underlying mechanisms of these chronic pain co-morbidities, but also for therapeutic purposes, as the specific treatment of one condition could have a significant impact upon the symptoms of the other. On this basis, the aim of the present study was to verify: firstly, if the association of FMS with migraine involves different levels of pain hypersensitivity with respect to one condition only; secondly if, in co-morbid patients, the hypersensitivity level is a function of migraine frequency; thirdly, if migraine attacks act as a triggering factor for FMS symptoms. Quantitative sensory tests were carried out in both painful and nonpainful sites and the correlation was explored between occurrence and frequency of migraine attacks and fibromyalgia exacerbations (flares). In addition, the effects were investigated of reducing migraine frequency via specific prophylaxis, on the degree of FMS pain and hyperalgesia.

Preliminary results have already been published in abstract forms [25, 26].

## Methods

The study was subdivided into two phases. The first phase evaluated the impact of migraine co-morbidity on somatic pain sensitivity and fibromyalgia pain. The second phase explored, in co-morbid patients, the effects of migraine prophylaxis on fibromyalgia symptoms. All patients attended the Headache and Fibromyalgia Center of the “G D’Annunzio” University of Chieti. The protocol adhered to the principles expressed in the Declaration of Helsinki and received ethic approval by the Institutional Review Board - Department of Medicine and Science of Aging – of the same University. A written informed consent was obtained from all patients (see inclusion criteria below).

### Phase 1

Patients affected with fibromyalgia and/or migraine were considered for the study, subdivided into five groups: a) fibromyalgia (FMS), b) high frequency episodic migraine (8–14 days/month)(M1); c) chronic migraine (≥15 days/month)(M2); d) fibromyalgia plus high frequency episodic migraine (FMS + M1); and e) fibromyalgia plus chronic migraine (FMS + M2). *Inclusion criteria for FMS* were: female sex; age 18–65 years; a diagnosis of fibromyalgia performed by a specialist 2–5 years previously (according to ACR 1990 criteria, with subsequent confirmation by 2010 criteria), with start of symptoms not before 6 years preceding their first visit to the Center, average intensity of diffuse musculoskeletal pain between 50 and 70 mm of VAS, under a stable dose of amitriptyline (10 mg/day) in the preceding 3 months (the vast majority of FMS patients attending our Center already are under some form of pharmacologic basal treatment for the continuous and intense nature of their condition. Since it would have been unethical to suspend it for the purpose of the study, we chose to minimize the effects of this treatment on the evaluated parameters by setting a homogeneous treatment regimen with low doses of amitriptyline, for all FMS groups, see below); exclusion of any concurrent pathology able to interfere with the sensory evaluation (e.g., hypertension, diabetes) [29, 30]; exclusion of any other chronic pain condition except fibromyalgia; a negative clinical history of any form of acute pain (except fibromyalgia flares) in the preceding 6 months; exclusion of major psychiatric disorders at specialistic psychiatric examination; written, informed consent to participate in the study.

*Inclusion criteria for M1 and M2* were: female sex, age 18–65 years, a diagnosis of migraine according to ICHD criteria (2004 criteria, confirmed by 2013 ICHD-3 beta criteria) [31, 32] with start of symptoms not before 12 years preceding the visit to the Center, and a number of migraine days ≥ 8 in the preceding 3 months; exclusion of any concurrent pathology able to interfere with the sensory evaluation (e.g., hypertension, diabetes); exclusion of any other chronic pain condition except migraine; a negative clinical history of any form of acute pain in the preceding 6 months; exclusion of major psychiatric disorders at specialistic psychiatric examination; informed, written consent to participate in the study.

*Inclusion criteria for FMS + M1 and FMS + M2* were: female sex; age 18–65 years; a diagnosis of fibromyalgia as for the FMS group and of migraine as for the M1 and M2 groups; exclusion of any other chronic pain condition except fibromyalgia and migraine; exclusion of any concurrent pathology able to interfere with the sensory evaluation (e.g., hypertension, diabetes); a negative clinical history of any form of acute pain in the preceding 6 months; exclusion of major psychiatric disorders at specialistic psychiatric examination; informed, written consent to participate in the study.

Out of a total of 325 examined patients, n. 203 meeting the inclusion criteria were selected: a) FMS (n. 40; 39.92 ± 6.17SD years); b) M1 (n.41; 38.15 ± 5.37 years); c) M2 (n. 40; 36.9 ± 5.61 years); d) FMS + M1 (n. 42; 38.74 ± 5.93 years); e) FMS + M2 (n.40; 37.17 ± 5.73 years). The five groups did not differ in mean age.

In all groups: pain thresholds to electrical stimulation in skin, subcutis and muscle were measured in multiple, nonpainful, body sites (deltoid, trapezius and quadriceps of one side)(control sites); muscle pressure pain thresholds were evaluated in the same locations and in the 18 TePs. Recording was also performed of: number of monthly migraine attacks in all migraine patients (3-month evaluation in an ad-hoc migraine diary); number of monthly fibromyalgia peak pain episodes (“flares”) in all FMS patients (3-month evaluation in an ad-hoc fibromyalgia diary); temporal relationship of FMS flares with migraine attacks in co-morbid groups (comparison of the two diaries; 3-month period).

### Phase 2

Patients affected with fibromyalgia plus high frequency episodic migraine (8-14 days/month) were considered (FMS + M1), subdivided into two groups: patients to be subjected to migraine prophylaxis (FMS + M1 *with* prophylaxis) and patients not to be subjected to prophylaxis (FMS + M1 *without* prophylaxis).

*Inclusion criteria for FMS + M1 with prophylaxis* were the same as for the FMS + M1 group of phase 1, plus: habitual acute medication for fibromyalgia flares represented by paracetamol 1 g; willingness to undergo migraine prophylaxis with calcium-channel blockers; no contraindications to treatment with this drug class [33, 34].

Calcium-channel blockers were selected as preventative treatment because they have no known direct influence on pain symptoms. Other preventative migraine options were discarded for different reasons: beta-blockers for their potential interference with cardiovascular reactivity, already particularly unstable in FMS patients e.g., postural hypotension, and antiepileptics for their direct impact onto pain perception. Further antidepressants were excluded since all patients were already under amitriptyline treatment. Among calcium-channel blockers, flunarizine was chosen as, according to guidelines, it represents the molecule with the highest level of evidence of efficacy [35–37].

*Inclusion criteria for FMS + M1 without prophylaxis* were the same as for the FMS + M1 group of phase 1, plus habitual acute medication for fibromyalgia flares represented by paracetamol 1 g; unwillingness to undergo any migraine prophylaxis in the immediate future (i.e., unwillingness to add a further prophylactic treatment for migraine to their already existing chronic treatment regimen with amitriptyline).

Out of 145 examined patients, n. 86 were selected who met the inclusion criteria: 47 patients for the treatment group (38.2 ± 6.2 years) and 39 for the non-treatment group (39.1 ± 5.1 years). The two groups did not differ in mean age. In basal conditions all patients underwent the following recordings: monthly number of migraine attacks and fibromyalgic flares (retrospective evaluation relative to the preceding 3 months); monthly analgesic consumption for the fibromyalgia flares (retrospective evaluation relative to the preceding 3 months); pain thresholds to electrical stimulation in skin, subcutis and muscle and pressure pain thresholds in multiple, nonpainful, body sites (deltoid, trapezius and quadriceps of one side)(control sites); pain thresholds to pressure stimulation at the 18 tender points (to calculate the mean threshold at TeP level). Patients of both groups were maintained on their standard chronic therapy for fibromyalgia with amitriptyline 10 mg/day. Patients of the FMS + M1 *with* prophylaxis group, but not those of the *without* prophylaxis group, were prescribed additional migraine prophylaxis with flunarizine (5 mg/die) for 3 months. Symptomatic treatment of fibromyalgia flares with paracetamol 1 g continued to be allowed.

All patients were requested to note, in ad-hoc diaries, the number of migraine attacks, fibromyalgia “flares” and rescue medications taken for fibromyalgia flares during the 3-month period of migraine prophylaxis, at the end of which pain thresholds were re-evaluated at both control areas and TeP level.

### Pain threshold assessment to pressure stimulation

A standard pressure dynamometer was used for the evaluation (Fischer’s algometer, Pain Diagnostic and Treatment, Inc., Great Neck, NY) [38]. The 1-cm diameter rounded probe of the instrument was placed perpendicularly on each evaluation site, pressure was increased by 0.1 kg-f/s until the first report of a painful sensation by the patient, the corresponding kg-f value was noted as the pressure pain threshold for that site. Thresholds were measured at the 18 TePs and in the trapezius, deltoid and quadriceps of one side (control sites). In each of these three muscles, two different points were evaluated (lateral and medial for trapezius – upper and lower for deltoid and quadriceps).

### Pain threshold assessment to electrical stimulation

A computerized constant current electrical stimulator (R.S.D. Stimulator, prototype, Florence 1997) delivered 18-ms trains of 0.5-ms monophasic square wave pulses, frequency 310 Hz, automatically every 2 s, to the skin via surface electrodes and to the subcutaneous tissue and the muscle through needle electrodes. Skin electrodes consisted of a 10-mm diameter circular plate in Ag/AgCl (reference electrode) and a cylinder in Ag/AgCl with a 0.3 mm-diameter base (stimulating electrode), placed 1 cm apart on the skin surface with interposition of conductor paste. Subcutis/muscle electrodes were two monopolar needles, 0.3 mm in diameter (teflon isolation except for 2 mm at the tip), inserted vertically, 1.5 cm apart, just below the skin surface for subcutis measurement and deep under the fascia for muscle measurement (intramuscular position verified by observing electrode movement under voluntary contraction and/or low-intensity electrical stimulation). Evaluated sites were the: lateral aspect of the upper border of the trapezius (not coinciding with the TeP site, electrodes placed in the horizontal direction), lower half of the deltoid and lowest third of the quadriceps (anterior aspect of the thigh). Measurement of thresholds in each tissue was performed by the method of the limits, to record typical pain sensations in each tissue (pricking pain for skin, linearly radiating prickling pain for subcutis, cramplike pain for muscle) according to a procedure already described in detail in previous publications [39–41].

Measurements were always performed at the same time of day (10:00– 12:00 a.m.), in the pain-free interval. During all measurements, patients lay comfortably on an adjustable examination bed in a quiet room. The experimenters evaluating thresholds were not aware of the group the patient belonged to.

### Statistical analysis

#### Phase 1

For each patient, the mean threshold was calculated of values recorded at the 18 TePs as the reference value for the specific FMS painful sites, and the mean threshold of the 6 values recorded in trapezius, deltoid and quadriceps as the reference value for the control sites. Means ± Standard Deviation (SD) were calculated of all parameters. Comparison among all groups for each parameter was performed via 1-way ANOVA.

To assess the temporal relationship between the occurrence of migraine attacks and fibromyalgic flares, for each patient the percentage was calculated of flares occurring within 12 h after a migraine attack vs occurrence of flares in any other period. Means ± SD were calculated of these percentages for each fibromyalgia patient group. The comparison between percentages of flares occurring within 12 h after a migraine attack and those of flares occurring at other time points in each group was performed via Wilcoxon matched-pairs signed-ranks test.

#### Phase 2

Means ± SD were calculated of all parameters. The comparison between treated and untreated groups was performed via Student’s *t*-test for unpaired samples. The comparison of parameters before and after prophylaxis in the treated group, and at comparable time points in the non-treated group, was performed via Student’s *t*-test for paired samples.

The level of significance was established at *p* < 0.05.

## Results and discussion

### Phase 1

#### Pain thresholds in control areas

**Fig. 1:**
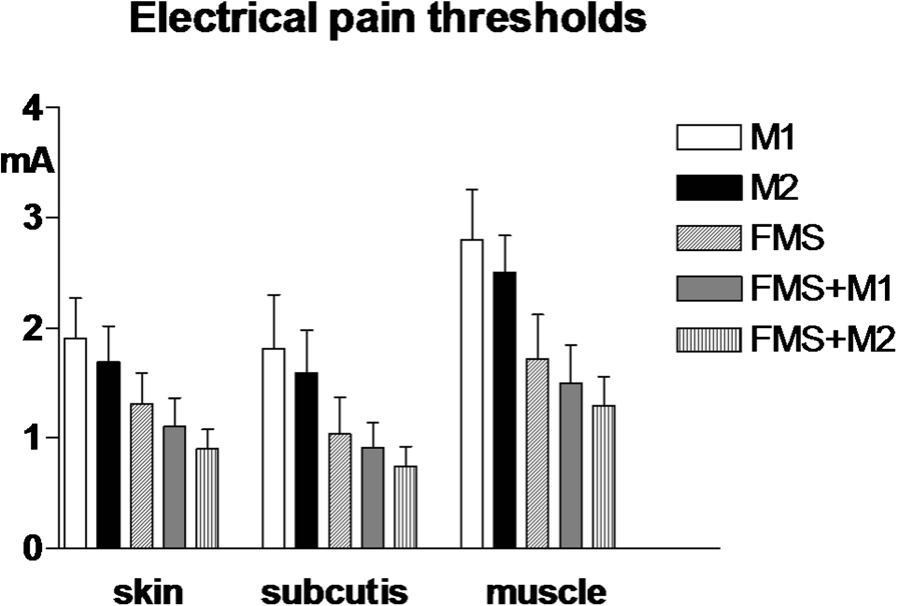
Pain thresholds to electrical stimulation in control areas (mean values recorded in trapezius, deltoid and quadriceps muscles and overlying subcutis and skin) in patients with high frequency episodic migraine (n. 41)(M1), chronic migraine (n.40)(M2), fibromyalgia (FMS)(n.40), fibromyalgia plus high frequency episodic migraine (n.42)(FMS + M1), fibromyalgia + chronic migraine (n.40)(FMS + M2)(Means ± SD). * = *p* < 0.05; ** = *p* < 0.01; *** = *p* < 0.001. 1-way ANOVA = significant trend for the three tissues, ***. Internal comparisons = for skin, M1 vs M2: **; M1 vs FMS, FMS + M1, FMS + M2: ***; M2 vs FMS, FMS + M1, FMS + M2: ***; FMS vs FMS + M1: *; FMS vs FMS + M2: ***; FMS + M1 vs FMS + M2: * for subcutis, M1 vs M2: *; M1 vs FMS, FMS + M1, FMS + M2: ***; M2 vs FMS, FMS + M1, FMS + M2: ***; FMS vs FMS + M2: *** for muscle, M1 vs M2: *; M1 vs FMS, FMS + M1, FMS + M2: ***; M2 vs FMS, FMS + M1, FMS + M2: ***; FMS vs FMS + M2: ***

**Fig. 2:**
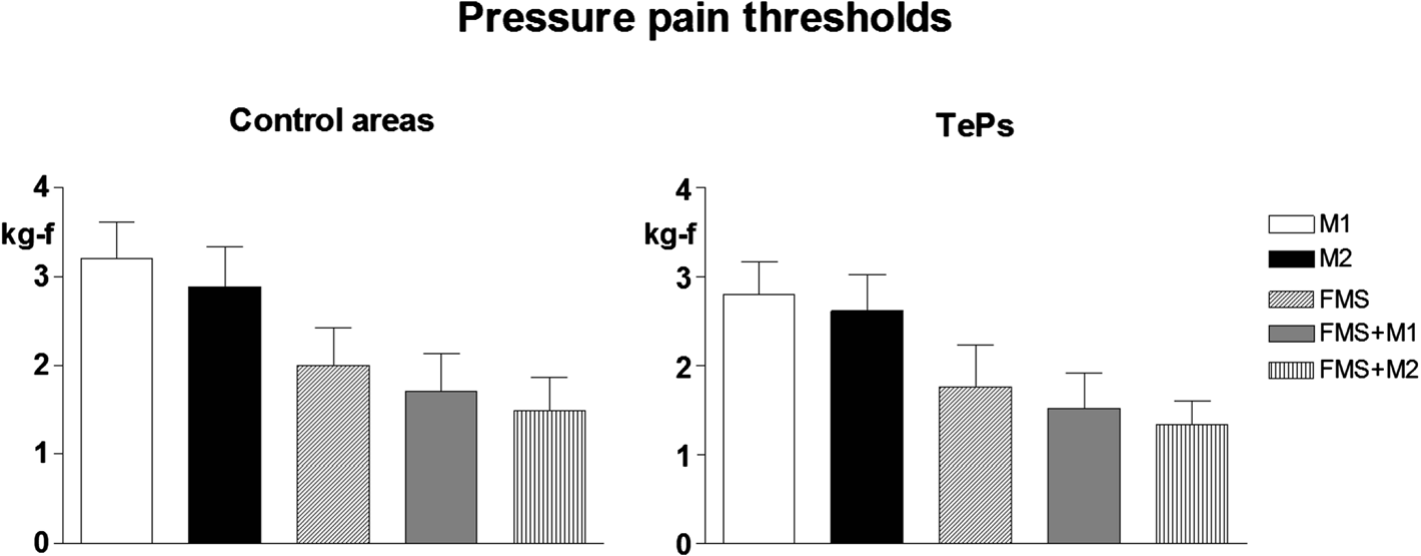
Pain thresholds to pressure stimulation in control areas (mean values recorded in trapezius, deltoid and quadriceps muscles and overlying subcutis and skin) (*left*) and in tender points (TePs) (mean values recorded at all 18 points)(*right*) in the same patient groups as for Fig. 1. 1-way ANOVA = significant trend for both control areas and TePs, ***. Internal comparisons for control areas = M1 vs M2: **; M1 vs FMS, FMS + M1 and FMS + M2: ***; M2 vs FMS, FMS + M1 and FMS + M2: ***; FMS vs FMS + M1: *; FMS vs FMS + M2:***. Internal comparisons for TePs = M1 vs FMS, FMS + M1 and FMS + M2: ***; M2 vs FMS, FMS + M1 and FMS + M2: ***; FMS vs FMS + M1: *; FMS vs FMS + H2:***

The lowest electrical thresholds at all body sites and all tissues and lowest muscle pressure pain thresholds were found in FMS + M2 followed by FMS + M1, FMS, M2 and M1. The trend for variation among groups was significant for all parameters (*p* < 0.0001) (Fig. 1 and Fig. 2 left).

#### Pain thresholds in TePs

The lowest mean pressure pain thresholds at tender point site were found in FMS + M2 followed by FMS + M1, FMS, M2 and M1. The trend for variation among groups was significant (*p* < 0.0001) (Fig. 2 right).

#### Fibromyalgia vs migraine symptoms

**Fig. 3:**
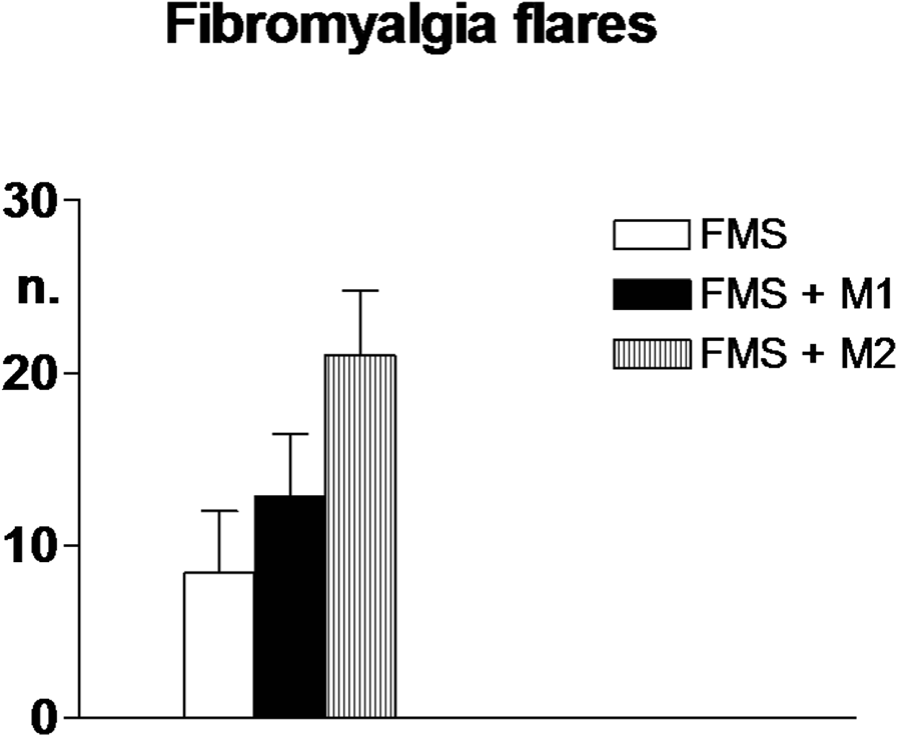
Mean monthly number of fibromyalgia flares in the three groups of fibromyalgia patients: fibromyalgia only (FMS; n. 40), fibromyalgia plus high frequency episodic migraine (FMS + M1, n. 42), fibromyalgia plus chronic migraine (FMS + M2, n. 40). Means ± SD. 1-way ANOVA = significant trend, ***. Internal comparisons = FMS vs FMS + M1 and FMS + M2: ***; FMS + M1 vs FMS + M2: ***

The mean number of monthly FMS flares was significantly and progressively higher in FMS, FMS + M1 and FMS + M2 groups (Fig. 3).

In co-morbid patients, the mean number of monthly migraine attacks was: 11.7 ± 2.1 in the FMS + H1 group and 19.4 ± 3.2 in the FMS + M2 group. The majority of FMS flares occurred within 12 h after a migraine attack, i.e., in 87 % of the cases in FMS + M1 and 86 % in FMS + M2. The difference with respect to flares occurring at a longer time distance from an attack was highly significant (*p* < 0.0001).

### Phase 2

#### Spontaneous pain

**Fig. 4:**
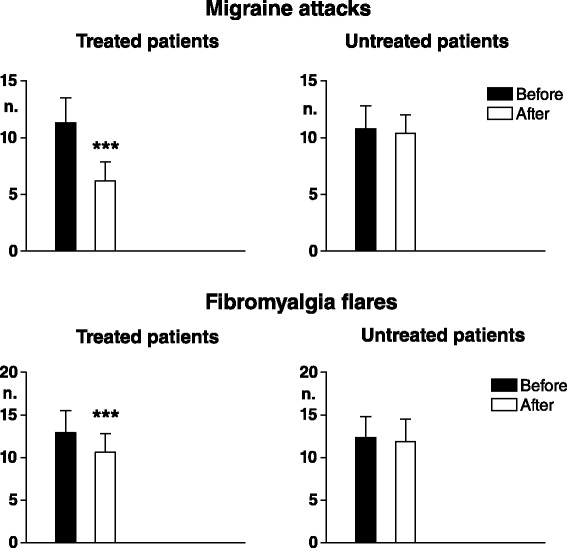
Mean monthly number of migraine attacks (*upper graphs*) and fibromyalgia flares (*lower graphs*) in two groups of patients with fibromyalgia plus high frequency episodic migraine (FMS + M1) undergoing migraine prophylaxis with flunarizine for 3 months (treated patients, n. 47) and not undergoing migraine prophylaxis (untreated patients, n. 39)(Means ± SD). Before: evaluation relative to the 3 months preceding start of treatment. After: evaluation relative to the 3 months of treatment. Asterisks above SD bars refer to comparison of before vs after values. *** = *p* < 0.001

**Fig. 5:**
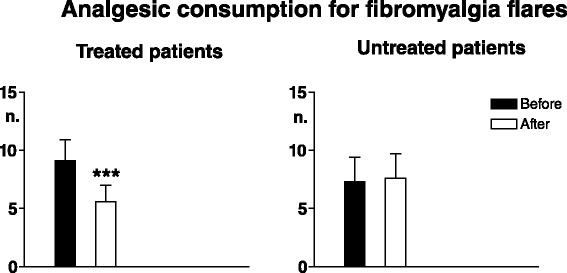
Mean monthly number of analgesic consumption for fibromyalgia flares. Legend as for Fig. 4

In patients undergoing migraine prophylaxis, the mean monthly number of migraine attacks was significantly reduced as compared with pre-treatment (*p* < 0.0001), while in patients not subjected to prophylaxis evaluated at comparable time points it did not vary significantly. In patients with prophylaxis but not in those without, also the mean monthly number of fibromyalgia flares and the number of rescue medications taken to treat them were significantly reduced (*p* < 0.0001) (Figs. 4 and 5).

#### Pain sensitivity

**Fig. 6:**
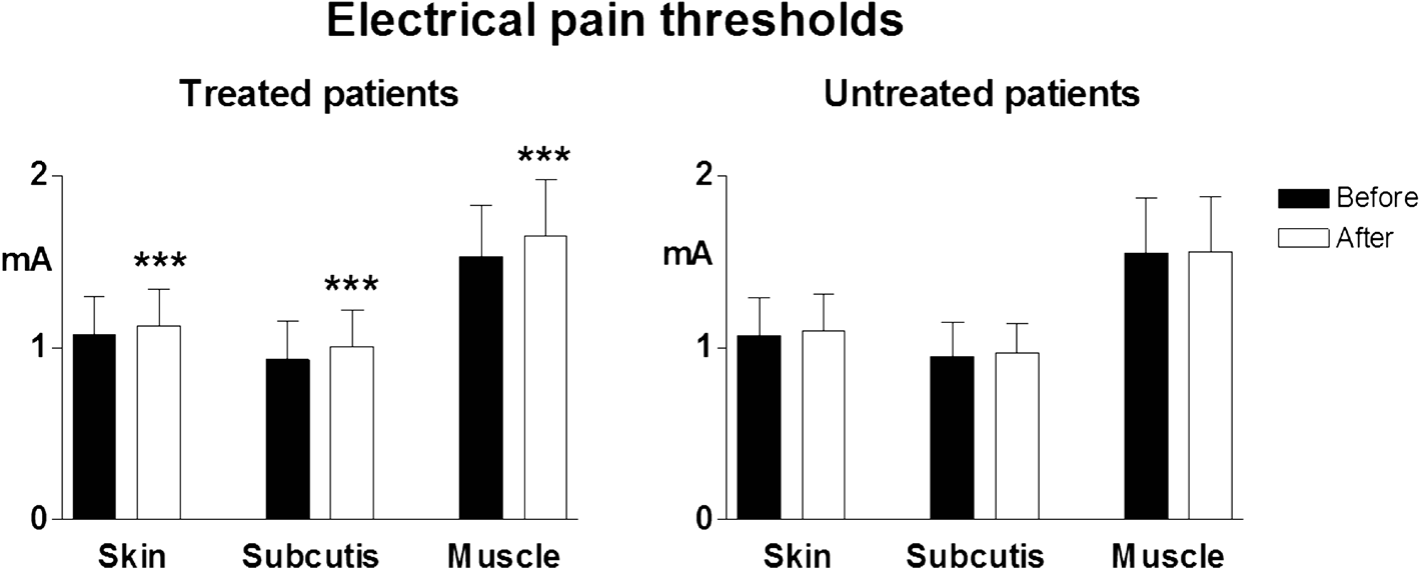
Pain thresholds to electrical stimulation in skin, subcutis and muscle in control areas. Legend as for Fig. 4

**Fig. 7:**
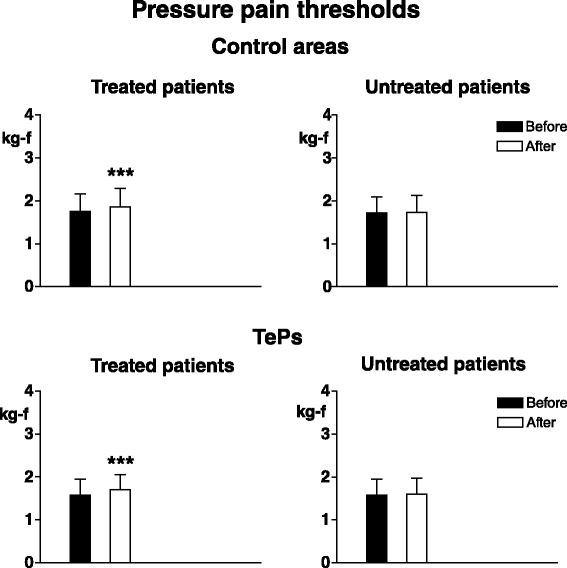
Pain thresholds to pressure stimulation in control areas (*upper graphs*) and in tender points (*lower graphs*). Legend as for Fig. 4

In treated patients electrical pain thresholds in control areas significantly increased in all tissues (*p* < 0.003 for skin, *p* < 0.0001 for subcutis and muscle) and pressure pain thresholds also significantly increased in both control areas (*p* < 0.0008) and TePs (*p* < 0.0005). No significant threshold changes were recorded in non-treated patients (Figs. 6 and 7).

A number of considerations can be made on the results of the study in regard to the co-morbidity of fibromyalgia with migraine. The first phase of the investigation shows firstly that the co-occurrence of both conditions involves a higher level of generalized hypersensitivity to pain at somatic level with respect to migraine only and fibromyalgia only, as testified by significantly lower pain thresholds in both painful and control areas. All FMS, but not non-FMS, patients were under stable basal amitriptyline treatment; though in very low doses, this treatment could have potentially influenced pain sensitivity in FMS, by increasing the pain threshold [35, 42]. However, even in this case, the outcome here found of a higher pain sensitivity in FMS vs non-FMS would not have changed without treatment; in contrast, the hypersensitivity difference would probably have been more pronounced.

The first phase of our study secondly shows that the hypersensitivity level is a function of the number of migraine attacks, with thresholds being lower in chronic than high frequency episodic migraine in both migraine-only patients and migraine patients plus fibromyalgia, confirming and extending previous results by de Tommaso et al. who showed that pain at tender points was significantly correlated with headache frequency in co-morbid patients [43].

Thirdly, in co-morbid patients it shows a high degree of temporal correlation between the occurrence of migraine and of FMS pain, with the vast majority of the fibromyalgia flares manifesting within 12 h after a migraine attack. On one hand these results reinforce the notion of a common pathophysiological mechanism in high frequency/chronic migraine and FMS, i.e., the central sensitization process. On the other hand they suggest that the algogenic input to the central nervous system due to the migraine attack is a triggering factor for a further enhancement of the existing hyperexcitability in the sensory compartment at the basis of fibromyalgia, similarly to what has already been documented for other pain generators in FMS, namely myofascial pain syndromes and painful joints [19, 44–46].

The second phase of the investigation further evidences the link between migraine and FMS symptoms. An effective reduction of the number of migraine attacks with a specific prophylaxis is, in fact, paralleled by a reduction also of the spontaneous (number of flares) and evoked (diffuse hyperalgesia) FMS symptoms. Such a result, obtained with a drug class (calcium-channel) not able to directly influence pain symptoms in fibromyalgia, with both patient groups under exactly the same stable dose of amitriptyline basal treatment, is probably due to the reduction of the central effect of nociceptive inputs from the cephalic area onto sensory neurons [33, 34]. Future additional studies will be necessary for confirmation, particularly as regards the effects of migraine prophylaxis, introducing a group of placebo-treated patients and a longer period of symptom assessment (beyond the 3-month duration of the migraine preventative regimen). To the best of our knowledge, however, the present investigation is the first to document the worsening impact of migraine pain on fibromyalgia symptoms in standardized conditions, suggesting that an effective control of the migraine co-morbidity is an important therapeutic measure also for FMS.

## Conclusions

In conclusion, co-morbidity between migraine and fibromyalgia involves a higher state of generalized somatic hypersensitivity towards painful stimuli with respect to one condition only. The increase in migraine frequency – with shift towards chronicity – promotes an enhancement of the hypersensitivity and of the spontaneous FMS pain.

These results suggest different levels of central sensitization in patients with migraine, fibromyalgia or both conditions and a role for migraine as a triggering factor for FMS. Prevention of headache chronification in migraine patients would thus appear crucial also for preventing the development of fibromyalgia in predisposed individuals or its worsening in co-morbid patients.

## Abbreviations

CNS: central nervous system
FMS: fibromyalgia
M1: high frequency episodic migraine
M2: chronic migraine
TePs: tender points

## References

[1] Hawkins RA (2013). Fibromyalgia: a clinical update. J Am Osteopath Assoc.

[2] Queiroz LP (2013). Worldwide epidemiology of fibromyalgia. Curr Pain Headache Rep.

[3] Wolfe F, Smythe HA, Yunus MB, Bennett RM, Bombardier C, Goldenberg DL, Tugwell P, Campbell SM, Abeles M, Clark P (1990). The American College of Rheumatology 1990 criteria for the classification of fibromyalgia. Report of the multicenter criteria committee. Arthritis Rheum.

[4] Wolfe F, Clauw DJ, Fitzcharles MA, Goldenberg DL, Katz RS, Mease P, Russell AS, Russell IJ, Winfield JB, Yunus MB (2010). The American College of Rheumatology preliminary diagnostic criteria for fibromyalgia and measurement of symptom severity. Arthritis Care Res (Hoboken).

[5] Bernik M, Sampaio TP, Gandarela L (2013). Fibromyalgia comorbid with anxiety disorders and depression: combined medical and psychological treatment. Curr Pain Headache Rep.

[6] Caldarella MP, Giamberardino MA, Sacco F, Affaitati G, Milano A, Lerza R, Balatsinou C, Laterza F, Pierdomenico SD, Cuccurullo F, Neri M (2006). Sensitivity disturbances in patients with irritable bowel syndrome and fibromyalgia. Am J Gastroenterol.

[7] Giamberardino MA, Affaitati G, Costantini R (2006). Chapter 24. Referred pain from internal organs. Handb Clin Neurol.

[8] Giamberardino MA, Affaitati G, Costantini R (2010). Visceral referred pain. J Musculoske Pain.

[9] Kato K, Sullivan PF, Evengård B, Pedersen NL (2006). Chronic widespread pain and its comorbidities: a population-based study. Arch Intern Med.

[10] de Tommaso M (2012). Prevalence, clinical features and potential therapies for fibromyalgia in primary headaches. Expert Rev Neurother.

[11] Sacco S, Ricci S, Carolei A (2011). Tension-type headache and systemic medical disorders. Curr Pain Headache Rep.

[12] de Tommaso M, Federici A, Loiacono A, Delussi M, Todarello O (2014). Personality profiles and coping styles in migraine patients with fibromyalgia comorbidity. Compr Psychiatry.

[13] Vij B, Whipple MO, Tepper SJ, Mohabbat AB, Stillman M, Vincent A (2015). Frequency of migraine headaches in patients with fibromyalgia. Headache.

[14] Küçükşen S, Genç E, Yılmaz H, Sallı A, Gezer İA, Karahan AY, Salbaş E, Cingöz HT, Nas Ö, Uğurlu H (2013). The prevalence of fibromyalgia and its relation with headache characteristics in episodic migraine. Clin Rheumatol.

[15] Marcus DA, Bhowmick A (2013). Fibromyalgia comorbidity in a community sample of adults with migraine. Clin Rheumatol.

[16] Abeles AM, Pillinger MH, Solitar BM, Abeles M (2007). Narrative review: the pathophysiology of fibromyalgia. Ann Int Med.

[17] Ablin JN, Buskila D (2015). Update on the genetics of the fibromyalgia syndrome. Best Pract Res Clin Rheumatol.

[18] Clauw DJ (2015). Fibromyalgia and related conditions. Mayo Clin Proc.

[19] Affaitati G, Costantini R, Fabrizio A, Lapenna D, Tafuri E, Giamberardino MA (2011). Effects of treatment of peripheral pain generators in fibromyalgia patients. Eur J Pain.

[20] Vecchiet L, Giamberardino MA, de Bigontina P, Dragani L, Gebhart GF, Hansmond DL, Jensen TS (1994). Comparative sensory evaluation of parietal tissues in painful and nonpainful areas in fibromyalgia and myofascial pain syndrome. Proceedings of the 7th world congress on pain, progress in pain research and management.

[21] Bezov D, Ashina S, Jensen R, Bendtsen L (2011). Pain perception studies in tension-type headache. Headache.

[22] Coppola G, Di Lorenzo C, Schoenen J, Pierelli F (2013). Habituation and sensitization in primary headaches. J Headache Pain.

[23] de Tommaso M, Sardaro M, Vecchio E, Serpino C, Stasi M, Ranieri M (2008). Central sensitisation phenomena in primary headaches: overview of a preventive therapeutic approach. CNS Neurol Disord Drug Targets.

[24] Filatova E, Latysheva N, Kurenkov A (2008). Evidence of persistent central sensitization in chronic headaches: a multi-method study. J Headache Pain.

[25] Giamberardino MA, Tafuri E, Di Fabio S, Tana C, Costa A, Fabrizio A, Affaitati G (2011). Chronic headache and fibromyalgia. J Headache Pain.

[26] Savini A, Tafuri E, Affaitati G, Fabrizio A, Lerza R, Sidonio G, Giamberardino MA (2008). Sensory evaluation in fibromyalgia and headache. J Headache Pain.

[27] Schmidt-Hansen PT, Svensson P, Bendtsen L, Graven-Nielsen T, Bach FW (2007). Increased muscle pain sensitivity in patients with tension-type headache. Pain.

[28] Zohsel K, Hohmeister J, Oelkers-Ax R, Flor H, Hermann C (2006). Quantitative sensory testing in children with migraine: preliminary evidence for enhanced sensitivity to painful stimuli especially in girls. Pain.

[29] Obrosova IG (2009). Diabetic painful and insensate neuropathy: pathogenesis and potential treatments. Neurotherapeutics.

[30] Viggiano A, Zagaria N, Passavanti MB, Pace MC, Paladini A, Aurilio C, Tedesco MA, Natale F, Calabrò R, Monda M, De Luca E (2009). New and low-cost auto-algometry for screening hypertension-associated hypoalgesia. Pain Pract.

[31] Headache Classification Subcommittee of the International Headache Society (2004). The international classification of headache disorders, 2nd edition. Cephalalgia.

[32] Headache Classification of the International Headache Society (2013). The international classification of headache disorders, 3rd edition (beta version). Cephalalgia.

[33] Giamberardino MA, Martelletti P (2015). Emerging drugs for migraine treatment. Expert Opin Emerg Drugs.

[34] Martelletti P (2015). The therapeutic armamentarium in migraine is quite elderly. Expert Opin Drug Metab Toxicol.

[35] Jagla G, Mika J, Makuch W, Obara I, Wordliczek J, Przewlocka B (2014). Analgesic effects of antidepressants alone and after their local co-administration with morphine in a rat model of neuropathic pain. Pharmacol Rep.

[36] Silberstein SD (2015). Preventive migraine treatment. Continuum (Minneap Minn). Headache.

[37] Sinclair AJ, Sturrock A, Davies B, Matharu M (2015). Headache management: pharmacological approaches. Pract Neurol.

[38] Fischer AA (1998). Muscle pain syndromes and fibromyalgia. Pressure algometry for quantification of diagnosis and treatment outcome. J Musculoskelet Pain.

[39] Giamberardino MA, Affaitati G, Lerza R, Lapenna D, Costantini R, Vecchiet L (2005). Relationship between pain symptoms and referred sensory and trophic changes in patients with gallbladder pathology. Pain.

[40] Giamberardino MA, Costantini R, Affaitati G, Fabrizio A, Lapenna D, Tafuri E, Mezzetti A (2010). Viscero-visceral hyperalgesia: characterization in different clinical models. Pain.

[41] Giamberardino MA, Tana C, Costantini R (2014). Pain thresholds in women with chronic pelvic pain. Curr Opin Obstet Gynecol.

[42] Ángel García D, Martínez Nicolás I, Saturno Hernández PJ. Clinical approach to fibromyalgia: synthesis of evidence-based recommendations, a systematic review. Reumatol Clin. 2015; [Epub ahead of print] 10.1016/j.reuma.2015.06.001 26481494

[43] de Tommaso M, Sardaro M, Serpino C, Costantini F, Vecchio E, Prudenzano MP, Lamberti P, Livrea P (2009). Fibromyalgia comorbidity in primary headaches. Cephalalgia.

[44] Gerwin R (2013). Are peripheral pain generators important in fibromyalgia and chronic widespread pain?. Pain Med.

[45] Giamberardino MA, Affaitati G, Fabrizio A, Costantini R (2011). Effects of treatment of myofascial trigger points on the pain of fibromyalgia. Curr Pain Headache Rep.

[46] Giamberardino MA, Affaitati G, Fabrizio A, Costantini R (2011). Myofascial pain syndromes and their evaluation. Best Pract Res Clin Rheumatol.

